# Phylogeography and taxonomic status of trout and salmon from the Ponto‐Caspian drainages, with inferences on European Brown Trout evolution and taxonomy

**DOI:** 10.1002/ece3.3884

**Published:** 2018-02-05

**Authors:** Levan Ninua, David Tarkhnishvili, Elguja Gvazava

**Affiliations:** ^1^ Institute of Ecology Ilia State University Tbilisi Georgia

**Keywords:** anadromous forms, brown trout, Ice Age, morphological evolution, phylogeography, Ponto‐Caspian region

## Abstract

Current taxonomy of western Eurasian trout leaves a number of questions open; it is not clear to what extent some species are distinct genetically and morphologically. The purpose of this paper was to explore phylogeography and species boundaries in freshwater and anadromous trout from the drainages of the Black and the Caspian Seas (Ponto‐Caspian). We studied morphology and mitochondrial phylogeny, combining samples from the western Caucasus within the potential range of five nominal species of trout that are thought to inhabit this region, and using the sequences available from GenBank. Our results suggest that the genetic diversity of trout in the Ponto‐Caspian region is best explained with the fragmentation of catchments. (1) All trout species from Ponto‐Caspian belong to the same mitochondrial clade, separated from the other trout since the Pleistocene; (2) the southeastern Black Sea area is the most likely place of diversification of this clade, which is closely related to the clades from Anatolia; (3) The species from the Black Sea and the Caspian Sea drainages are monophyletic; (4) except for the basal lineage of the Ponto‐Caspian clade, *Salmo rizeensis*, all the lineages produce anadromous forms; (5) genetic diversification within the Ponto‐Caspian clade is related to Pleistocene glacial waves; (6) the described morphological differences between the species are not fully diagnostic, and some earlier described differences depend on body size; the differences between freshwater and marine forms exceed those between the different lineages. We suggest a conservative taxonomic approach, using the names *S. rizeensis* and *Salmo labrax* for trout from the Black Sea basin and *Salmo caspius* and *Salmo ciscaucasicus* for the fish from the Caspian basin.

## 1 INTRODUCTION

Until recently, brown trout (*Salmo trutta* sensu Lato) was considered to be a widespread western Eurasian fish species with facultative anadromy. Its natural range stretches from West Siberia to the Atlantic and throughout West Asia (Maitland & Linsell, 2006). Simultaneously, the taxonomy of brown trout is controversial. In older treatises (e.g., Sabaneev, 1875), it is separated into purely freshwater forms *S. t. lacustris* and *S. t. fario*, and the anadromous *S. t. trutta*. Anadromy may not be a heritable character for brown trout (Berg, 1959, 1962); hence, these names probably do not have any taxonomic meaning. Fishbase (www.fishbase.org), considering recent changes in taxonomy, lists more than 20 species that formerly were qualified as geographic populations or subspecies of *S. trutta*. Eight of those are potentially present in the Caucasus Ecoregion (as defined in Zazanashvili, Sanadiradze, Bukhnikashvili, Kandaurov, & Tarkhnishvili, 2004) and, broader, in the basins of the Black and the Caspian Seas (from here onwards—Ponto‐Caspian Basin: the drainages of the Black and the Caspian Seas formed a contiguous body of water separated from the Mediterranean in the geological past—Popov et al., 2004). *Salmo ciscaucasicus* (Dorofeeva, 1967; syn. *S. trutta ciscaucasicus*) is native for the northwestern drainages of Caspian Sea, including the basin of the Terek (Tergi) River (Kottelat & Freyhof, 2007). *Salmo caspius* (Kessler, 1877; syn. *S. trutta caspius*) is native to the southern Caspian and the rivers of the northern Iran (Turan, Kottelat, & Engin, 2009). *Salmo coruhensis* (Turan et al., 2009) is an anadromous form from the southeastern and, possibly, eastern Black Sea basin in Turkey and Georgia. *Salmo rizeensis* (Turan et al., 2009) is a riverine form from the same area and other rivers of the southern Black Sea basin. *Salmo ischchan* (Kessler, 1877) is a lacustrine form from the Lake Sevan in Armenia (Berg, 1962). *Salmo ezenami* (Berg, 1962; syn. *S. trutta ezenami*) inhabits Lake Kezenoi‐Am in the northeastern Caucasus (Bogutskaya & Naseka, 2002). “Black Sea Salmon” (*Salmo labrax* Pallas, 1814) is an anadromous fish reproducing in the rivers draining into the Black Sea (Kottelat & Freyhof, 2007). Lastly, European brown trout (*Salmo trutta* Linneaus, 1758) is also found in some rivers of the Caspian and the Black Sea basins (Svetovidov, 1984).

Genetic and/or morphological and/or geographic distinctiveness was not demonstrated sufficiently well for some of these species. Turan et al. (2009) showed nearly fixed morphological and genetic differences between *S. coruhensis* and *S. rizeensis* from the southeastern Black Sea drainage. The individuals of *S. labrax* from the northern Black Sea drainage, described in the same paper, differ morphologically from another anadromous form, nominal *S. coruhensis*, although the differences are not fully diagnostic and the individuals were not studied genetically. It is not clear whether there are fixed differences between *S. labrax* and *S. trutta* from the rivers draining into the Black Sea from the north and the west, although some differences are mentioned (Kottelat & Freyhof, 2007). Fixed differences are suggested between *S. caspius* and *S. labrax* in the number of gill rakers (Turan et al., 2009); however, the studied individuals of *S. caspius* were smaller than those of the latter, and the influence of size on morphology cannot be excluded a priori. Moreover, the described *S. caspius* individuals were not studied genetically.

One could expect that speciation in brown trout should follow geological patterns, that is, catchments separated later should have more closely related trout lineages. This can be used as a null hypothesis challenged by the observed taxonomic diversity, which assumes the presence of more than one species in the same catchment: *S. coruhensis*,* S. rizeenzis,* but also potentially *S. labrax* in the western drainage of the Black Sea; *S. ischchan* and *S. caspius* in the Kura‐Aras catchment (Lake Sevan is connected with the Aras River through the River Razdan); *S. labrax* and *S. trutta* in central and eastern European rivers.

Aiming to clear up these taxonomic and evolutionary puzzles and to infer which nominal species of brown trout are present throughout the Caucasus Ecoregion, the authors collected samples from six river drainages in Georgia, including four flowing into the Black Sea and two into the Caspian, we characterized external morphology of these fish, and analyzed their mitochondrial haplotypes, along with the haplotypes of brown trout from different sea and river drainages available from GenBank.

## 2 MATERIAL AND METHODS

### 2.1 Sampling

**Figure 1:**
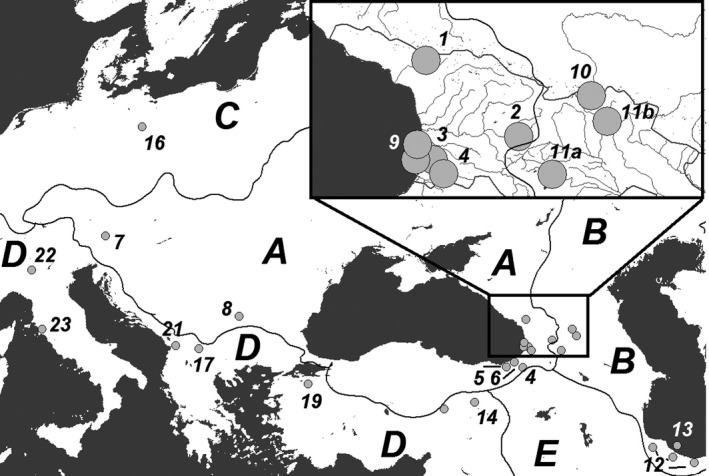
Sampling locations, according to our data (1–4, 9–11) and existing publications (see Table S1 for the location/publication list). The numbers correspond to the location names listed in Table 1. Catchments: *A*—Black Sea, *B—*Caspian Sea, *C—*Atlantic Ocean/Baltic Sea, *D*—Mediterranean Sea, *E*—Persian Gulf/Indian Ocean

The sampling locations (and exact or approximate locations of fish for sequences that were downloaded from Genbank) are shown in Figure 1. The total number of samples used in genetic and morphological study is shown in Table 1. Fish were caught by netting, using the net with the diameter of 1.5 cm from August 2014 through November 2014. Permits were obtained from Ministry of Environment and Natural Resources Protection of Georgia (permit # 4029, 21/07/2014). Pictures of caught fish were taken for morphometric study. Samples of tissue (fin clips) were stored in 95% ethanol for subsequent DNA analysis.

**Table 1:** The number of samples used for the morphometric and genetic analysis original samples and downloaded sequences) from individual sea and river catchments

Drainage, Basin	Species of *Salmo* as referred	Morphology	Cyt *b*	Control region
Kura (Mtkvari) (CA)	*Salmo* sp.^a^	8	6	4
Terek (Tergi) (CA)	*Salmo* sp.^a^	8	6	7
Enguri (BS)	*Salmo* sp.^a^	12	5	
Rioni (BS)	*Salmo* sp.^a^		7	3
Kintrishi (BS)	*Salmo* sp.^a^		10	6
Coruh (Chorokhi) (BS)	*Salmo* sp.^a^	6	6 + 3^b^	6
Black Sea (Georgia)	*Salmo* sp.^a^	9	8	
Danube (BS)	*S. trutta*		10^b^	11^b^
Plitvica (BS)	*S. trutta*			1^b^
Aral Sea	*S. trutta oxianus*			2^b^
Caspian Sea (CA)	*S. caspius*		1^b^	
Arpa (CA)	*S. caspius*		1^b^	
Rivers of southern Caspian (CA)	*S. caspius*		2^b^	11^b^
Iyidere (BS)	*S. coruhensis*		2^b^	
Iyidere (BS)	*S. rizeensis*		1^b^	
Cayeli (BS)	*S. rizeensis*		2^b^	
Turkey (not specified)	*S. trutta*			11^b^
Soguksu Stream (ME)	*S. platycephalus*		1^b^	
Stilaro (ME)	*S. trutta*			1^b^
Göksu River (ME)	*S. trutta*		1^b^	
Lake Ohrid (AD)	*S. ohridanus*		1^b^	
Zala (AD)	*S. marmoratus*		1^b^	
Buna (AD)	*S. obtusirostris*		1^b^	
Shkumbini (AD)	*S. trutta*			1^b^
Seta (AD)	*S. trutta*			1^b^
Voidomatis (AD)	*S. trutta*		1^b^	
Leksa (AT)	*S. trutta*		1^b^	
Ims (AT)	*S. salar*		1^b^	
Dades (AT)	*S. trutta*		1^b^	
Tensift (AT)	*S. trutta*		1^b^	
Oum er Rbia (AT)	*S. trutta*		3^b^	
Moulouya (AT)	*S. trutta*		1^b^	
Isli Lake (AO)	*S. trutta*		1^b^	
Ifni Lake (AO)	*S. akairos*		2^b^	
Atlantic Ocean (AO)	*S. salar*			1^b^
North Sea (AO)	*S. trutta*		1^b^	
Baltic Sea (AO)	*S. trutta*		2^b^	
Baltic Sea (AO)	*S. salar*		1^b^	
Euphrates (IO)	*S. trutta* c		2^b^	
Indian Ocean Basin (Introduced)	*S. trutta*		5^b^	

BS, Black Sea drainage; CA, Caspian Sea drainage; PG, drainage of Persian Gulf; ME, Mediterranean Sea drainage; AO, drainage of the Atlantic Ocean.

a *Salmo labrax* or *S. coruhensis* or *S. rizeensis*.

b Sequences downloaded from GenBank.

c According to Turan et al. (2009).

### 2.2 DNA extraction, PCR, and sequencing

DNA was extracted from gill or muscle tissue of trout using the Qiagen DNeasy tissue kit according to the manufacturer's instructions. A mitochondrial cytochrome *b* gene fragment was amplified using the following primers: nSsaL14437 (5′‐GCTAATGACGCACTAGTCG‐3′) (Warheit & Bowman, 2008) and StrCBR (5′‐GGGGGCGAGRACTAGGAAGAT‐3′) (Turan et al., 2009). Mitochondrial control region was amplified with primers BrtD‐F20 (5′‐GAGATTTTAACTCCCACCCT‐3′) and BrtD‐R20 (5′‐TAGGGTCCATCTTAACAGCT‐3′) (Segherloo, Farahmand, Abdoli, & Bernatchez, 2012). Amplification conditions were the same for both mitochondrial fragments. Twenty microlitre PCR reactions contained 3 μl of template DNA, 1U Promega Tag polymerase, 1X promega buffer, 2.5 mmol/L MgCl_2_, 0.1 mmol/L of each dNTPs, and primer concentrations 0.1 μmol/L. Thermal profile was as follows: 3‐min initial denaturation at 94°C, followed by 35 cycles at 94°C for 40 s, 50°C for 1 min, and 72°C for 2 min, and 10 min at 72°C for final extension. 5 μl from each PCR was run on 1% agarose gel to visualize the DNA fragments. The amplicons were sequenced on an ABI 3130 Gene Analyser. PCR fragments were sequenced in both directions using the same PCR primers and Big Dye Terminator 3.1, as per manufacturer's protocol. The *unique* sequences of the mitochondrial cytochrome *b* gene and that of the mitochondrial control region from our samples were deposited to GenBank (accession # MG029536–MG029553 for Cyt‐*b* and MG214765–MG214775 for the control region).

### 2.3 Sequence analysis

We analyzed an 842‐bp‐long region of the mitochondrial cytochrome *b* (48 samples and 27 sequences downloaded from GenBank) and a 505‐bp‐long control region fragment (26 samples and 37 sequences downloaded from GenBank, Table 1 and Table S1). Unfortunately, the cytochrome *b* and control region sequences downloaded from GenBank were from different publications and described different individuals and populations; hence, we did not concatenate the studied fragments while inferring phylogenies and conducted the analyses separately for these two fragments of mitochondrial DNA.

We used three methods for the analysis of cytochrome *b* sequences: (1) Minimum spanning network was constructed only for the trout from the rivers drawing into the Black and the Caspian Seas (Ponto‐Caspian Basin) using NETWORK 5.0 (Bandelt, Forster, & Rohl, 1999). (2) Maximum likelihood (ML) tree was built and bootstrap support was estimated using software MEGA 7.0.21 (Kumar, Stecher, & Tamura, 2016). In this analysis, besides the fish from the Ponto‐Caspian drainage, the fish from the drainage of the Indian and the Atlantic Oceans and Mediterranean (nominal *S. trutta*,* S. marmoratus*,* S. platycephalus,* and *S. akairos*) were included along with Balkan species *S. ohridanus* and *S. obtusirostris* (Crête‐Lafrenière, Weir, & Bernatchez, 2012), and Atlantic salmon (*S. salar*) was added to the analysis as the outgroup. For this analysis, only one haplotype/location was included if more than one individual from the same location had the same haplotype. The best‐fit model (that with the lowest Bayesian Information Criterion, BIC, as recommended in Nei & Kumar, 2000) was identified with MEGA 7.0.21 using ML method (3) Bayesian inference (BI) tree was built using the same taxa included in the ML analysis, for validating the tree topology using different approach, counting posterior probability of the branches, and inferring split times using BEAST v. 1.8.4 (Drummond, Suchard, Xie, & Rambaut, 2012). The Bayesian analysis was initiated from random starting trees, assuming uncorrelated log‐normal relaxed clock model and a coalescent model with constant population size. Posterior distributions of parameters were approximated using Markov chain Monte Carlo with chain length set at 100,000,000 to provide sufficient sample size for each parameter (i.e., effective sample size ≫ 100). The same substitution model was used as in the ML analysis.

We inferred the best‐fit model and conducted ML analysis for the obtained and downloaded sequences of control region (see Table 1 for the list of the individuals used in this analysis). These sequences did not show much informative variability among the studied taxa, and we did not apply BI for this dataset. The software used was MEGA 7.0.21.

We used BEAST v. 1.8.4 (the uncorrelated log‐normal relaxed molecular clock model) to account for variable rates of evolution among the lineages and to infer the 95% HPD intervals for the node ages, based on cytochrome *b* gene variation. For calibrating the tree, we used an estimated time of split between the reference cytochrome *b* sequences included in our analysis, drawn from multiple publications (www.timetree.org; Hedges, Marin, Suleski, Paymer, & Kumar, 2015). The divergence time between *S. ohridanus* and *S. obtusirostris*, in one clade, and *S. marmoratus*,* S. platycephalus*, and *S. trutta* in the other was set as a time of the initial split.

We also used the alternative scaling by Schenekar, Lerceteau‐Köhler, and Weiss (2014), not considered by www.timetree.org, and based on the sequence analysis of two distant lineages (“Atlantic” vs. “Black Sea” lineages) from Austria (Schenekar et al., 2014), which suggests a much later split between the lineages than other authors (Alexandrou, Swartz, Matzke, & Oakley, 2013; Crête‐Lafrenière et al., 2012; Macqueen & Johnston, 2014).

### 2.4 Morphometry

**Figure 2:**
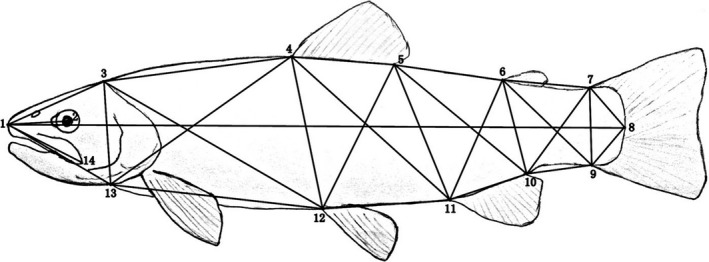
Landmarks (1–14) and the distances between the landmarks using in the morphometric analysis of brown trout and salmon used in the analysis

Fish caught in Georgia were photographed from the lateral side. The images were used for scoring the 28 conventional distances among the 14 landmarks shown in Figure 2, using software ImageJ (Rasband, 2016). The distances were log‐transformed to control for allometric effects. Subsequently, the regression of each of the 27 distances on the first distance (landmarks 1–8, reflecting body length of an individual) was calculated and the standardized residuals on the regression line (*size‐removed* body proportions) were used for final calculations as recommended by Thorpe and Leamy (1983), instead of the original 28 distances. Principal component analysis (PCA) was applied for extracting principal components with loadings exceeding unity, and for calculating the individual scores. The individual scores on PCA loadings 1–4 were used for ordination of the individuals and inferring possible differences among the species, which revealed genetic groups, and life forms (riverine vs. marine). All calculations were conducted using SPSS 21.0 for Windows. River trout from Georgia was included in the analysis along with the marine form (“Black Sea salmon”), and two individuals of North American rainbow trout (*Onychorhynus mykiss*), an introduced/invasive species in Georgia's rivers and lakes.

Besides this morphometric study, we counted (1) the number of rays in dorsal fins, (2) the number of main rays in pectoral fins, and (3), the number of gill rakers at the first gill arch (Andersson, Johansson, Sundbom, Ryman, & Laikre, 2016; Kara, Alp, & Gürlek, 2011).

## 3 RESULTS

### 3.1 Minimum spanning network

**Figure 3:**
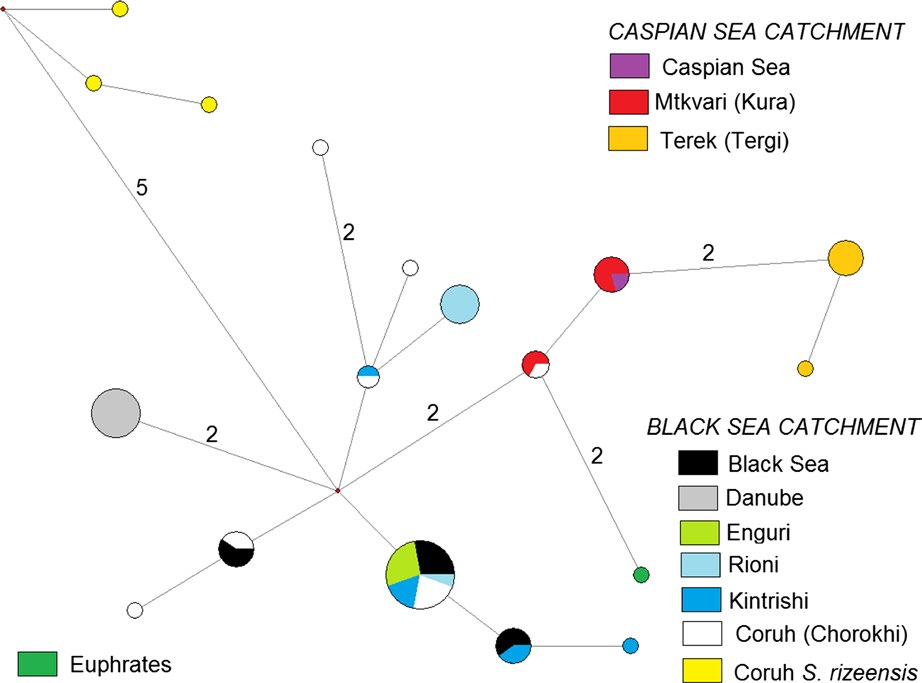
Median‐joining network showing the revealed cytochrome *b* haplotypes of trout and salmon from the Ponto‐Caspian basin. Size of pies proportional to the number of fish with a specific haplotype among studied specimens. Numbers at the lines—the number of substitutions separating the haplotypes if exceeding one). The colors show drainages of different rivers/seas. *S. rizeensis is* shown in color different from other fish from Coruh (Chorokhi) drainage. “Mtkvari,” “Terek,” and “Caspian Sea” belong to the Caspian Sea drainage, “Euphrates”—to the drainage of Persian Gulf, and all others—to the Black Sea drainage

Seventeen haplotypes were revealed among the Cyt‐*b* sequences of brown trout and salmon from the Ponto‐Caspian drainages, including those obtained from our samples and those downloaded from Genbank (Figure 3). The sequences of *S. rizeensis* from the paper of Turan et al. (2009) were the most distant from the others and had at least five substitutions that separate them from the next closest haplotypes. The rest of the sequences is subdivided into those from the Black Sea (from the catchments of the Rivers Coruh (Chorokhi) with its tributaries Ajaristskali, Kintrishi, Rioni, Enguri, the Danube, the coast of the Black Sea in Georgia) and those from the Caspian Sea drainage (from the catchments of the Rivers Tergi (Terek), Mtkvari (Kura) with its tributaries Ktsia and Aragvi, and coast of the Caspian Sea in Iran) (Figure 1). There were a maximum of 10 substitutions between the most distant haplotypes from the Black Sea basin, and a maximum of four substitutions between the haplotypes from the Caspian Sea basin. A single individual with the “Caspian” haplogroup was found in drainage of Coruh, and one also from the Euphrates (Figure 3).

### 3.2 Phylogeny based on cytochrome‐*b* sequences

**Figure 4:**
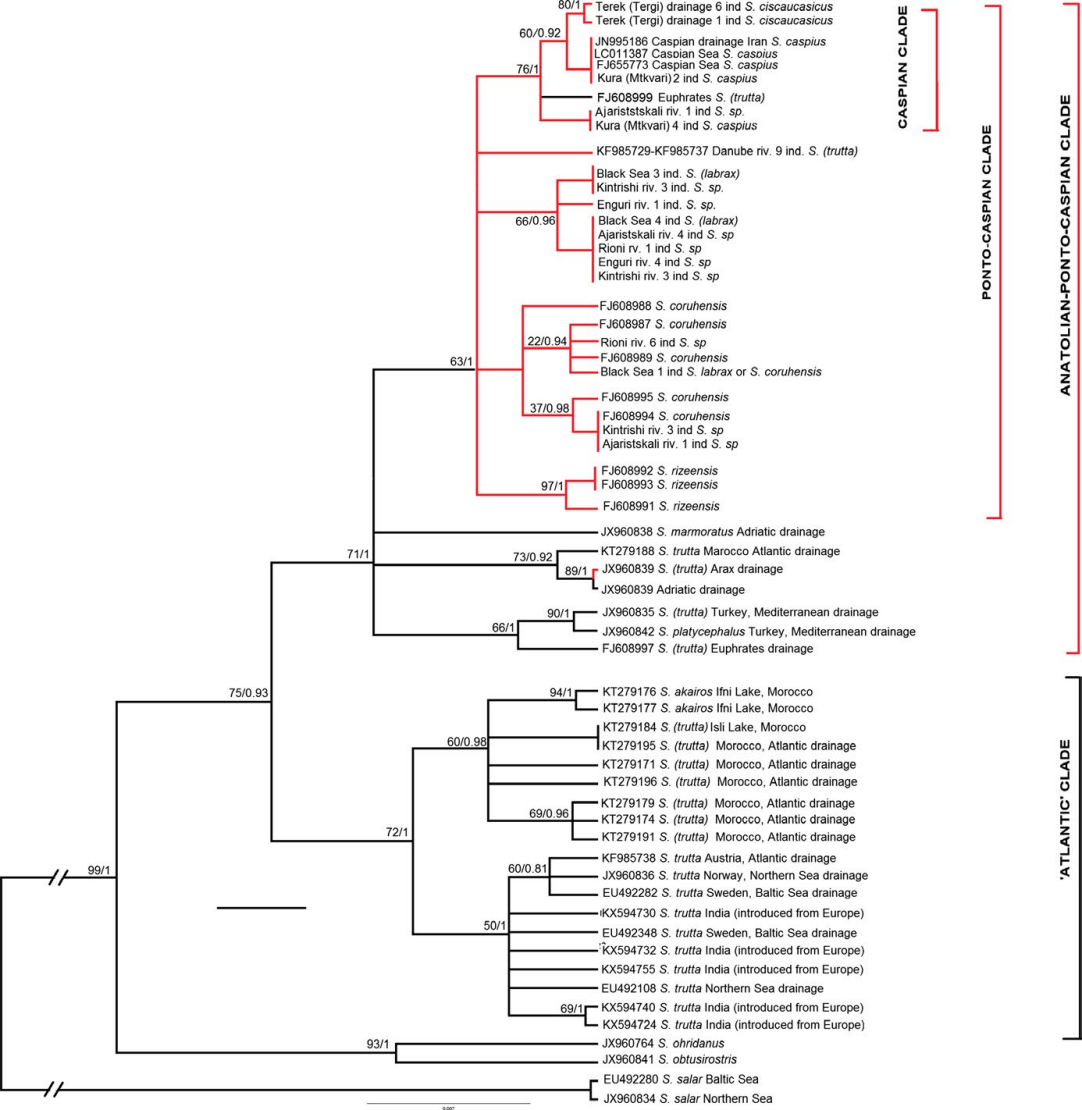
Phylogeny of the brown trout/salmon from the Ponto‐Caspian basin, Anatolia/basin of the Indian Ocean, basin of Atlantic Ocean, and the Balkans, based on the mitochondrial cytochrome *b* gene sequences. The topology inferred using Bayesian analysis (BI). Left to the nodes: bootstrap support of the respective clade inferred using maximum likelihood (ML) algorithm before the slash, posterior probability as inferred by BI after the slash. Identical haplotypes shown separately if found at more than one location. Clades with bootstrap support below 50 and PPs below 0.5 shown as polytomies. Red branches—samples from the drainage of the Black Sea; blue branches—samples from the drainage of the Caspian Sea; green branches—samples from the Atlantic, Northern, and Baltic Sea drainages

The optimal substitution model for the ingroups included in the analysis was *TN93+G* (Kumar et al., 2016). Both ML and Bayesian analyses suggested the presence of 22 well‐supported major mitochondrial clades within the analyzed sequences (Figure 4). The topology of the tree suggests that: (1) Trout form a monophyletic clade including “Balkan” species (*S. ohridanus + S. obtusirostris*). (2) All trout sequences of cytochrome *b* gene, including new sequences from this research and those available from the GenBank, excluding *S. ohridanus* and *S. obtusirostris* (which are sister species) form a monophyletic clade. (3) All trout sequences of the cytochrome *b* gene, excluding *S. ohridanus* and *S. obtusirostris,* are subdivided into two monophyletic clades: one from the rivers flowing into the Atlantic Ocean (including trout of European origin introduced to India), and the other comprised of fish from the Mediterranean and Ponto‐Caspian drainages, including fish from the Euphrates River. (4) Almost all (68 of 69) samples from the rivers and lakes of the Ponto‐Caspian drainages, and from the upper Danube in Austria and the rivers flowing into the Caspian Sea in Iran, including Black Sea salmon and Caspian salmon, belong to a monophyletic mitochondrial clade, nested into the “Mediterranean‐West Asian” clade (hereafter Ponto‐Caspian clade); (5) The Ponto‐Caspian clade is subdivided into five monophyletic haplotypes and/or haplogroups: one (with two exceptions) from the Caspian Sea drainage, one from Danube River basin, and three from the eastern and southeastern Black Sea drainage, including nominal *S. rizeensis,* which belongs to a separate monophyletic clade. (6) The clades from the eastern Black Sea drainage, except *S. rizeensis* clade, are not geographically distinct. One of these clades was found in catchments of the Rivers Coruh (Chorokhi), Rioni, and in the Black Sea; in the catchments of the Rivers Coruh (Chorokhi), Kintrishi, and in the Black Sea; in the catchments of the Rivers Coruh (Chorokhi), Kintrishi, Rioni, Enguri, and in the coastal area of the Black Sea. The trout individuals identified as *S. coruhensis* by Turan et al. (2009) were present in both clades.

We conclude that anadromous life mode is typical for all trout lineages from the eastern Black Sea drainage, with the exception of *S. rizeensis* from the northeastern Turkey. The same is true for the clade from the Caspian Sea drainage, which contains at least one salmon captured in the Caspian Sea. This clade contains, additionally, a well‐supported subclade of fish from the basin of the River Terek (Tergi) (nominal *S. ciscaucasicus*). Another important conclusion is that the basal genetic diversification of brown trout from the Ponto‐Caspian region occurred in the northern and eastern Anatolia, where the split between the Mediterranean and Ponto‐Caspian lineages most likely occurred.

### 3.3 Inferred time of split among the clades

**Figure 5:**
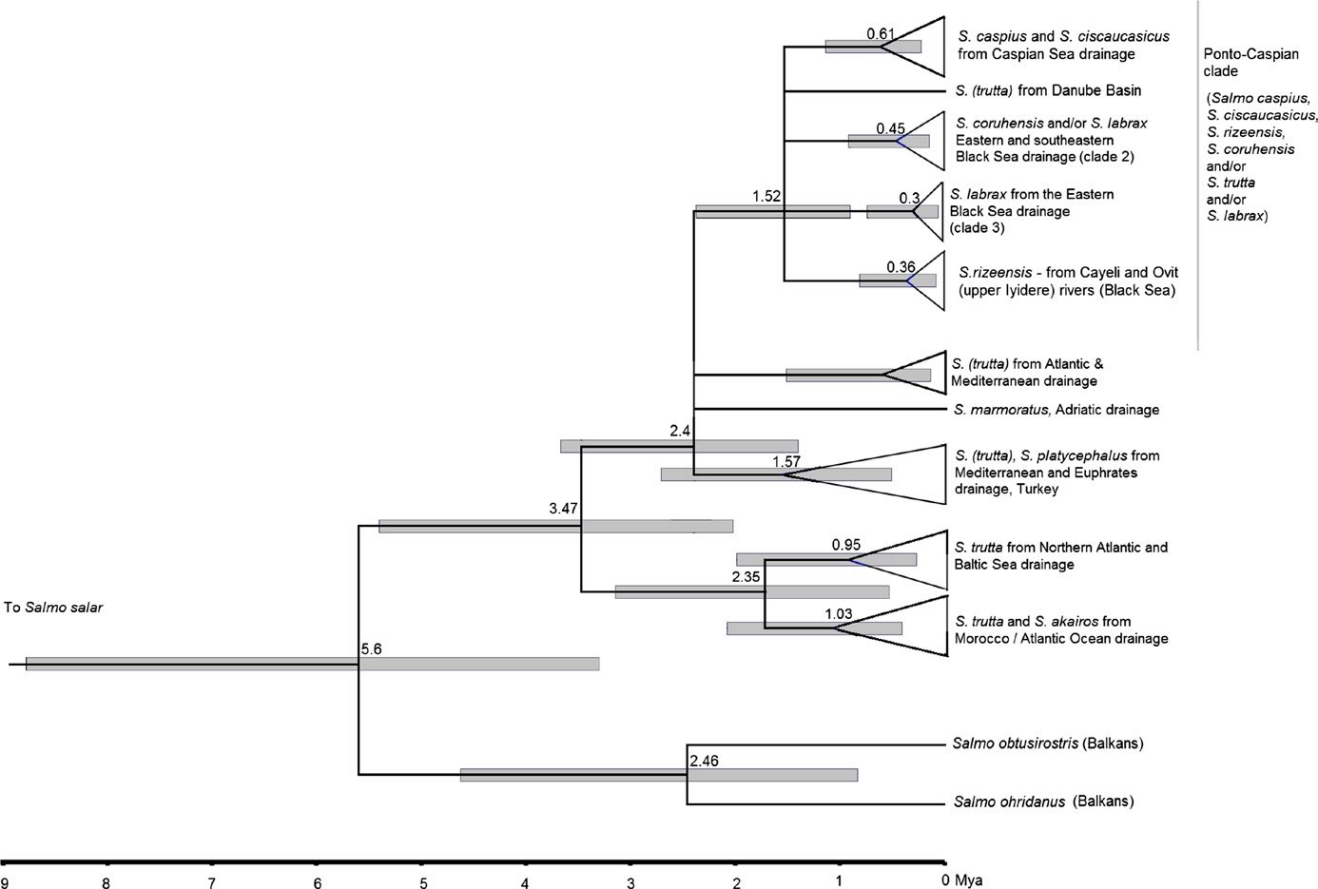
Timescale for the divergence of the clades shown in Figure 2, according to the consensus calibration of www.timetree.org. The numbers at the nodes—estimated divergence time (millions of years, mya); gray horizontal bars—95% HPD intervals

If the time of split between *S. ohridanus* and *S. obtusirostris,* on one side, and *S. marmoratus, S. platycephalus,* and *S. trutta* on the other (5.6 mya) is set as a time of the initial split, the divergence among the clades is dated as shown in Figure 5. The split between the brown trout from the Atlantic drainage and Anatolian/Mediterranean/Ponto‐Caspian lineage occurred 3.5 (range 2.1–5.4) mya; the split between the Mediterranean/Euphrates basin and the Ponto‐Caspian lineage occurred 2.4 (1.9–3.6) mya; the split between the five subclades of the Ponto‐Caspian brown trout—1.5 (0.9–2.4) mya.

If calibration used by Schenekar et al. (2014) is applied, these figures should be corrected. The average split time between the subclades of the Ponto‐Caspian clade is in this case only ca. 230 kya. This does not affect the main conclusion: The earliest genetic diversification of brown trout from the Ponto‐Caspian Basin occurred at the southeastern coast of the Black Sea and the rivers located in this area.

### 3.4 Control region

**Figure 6:**
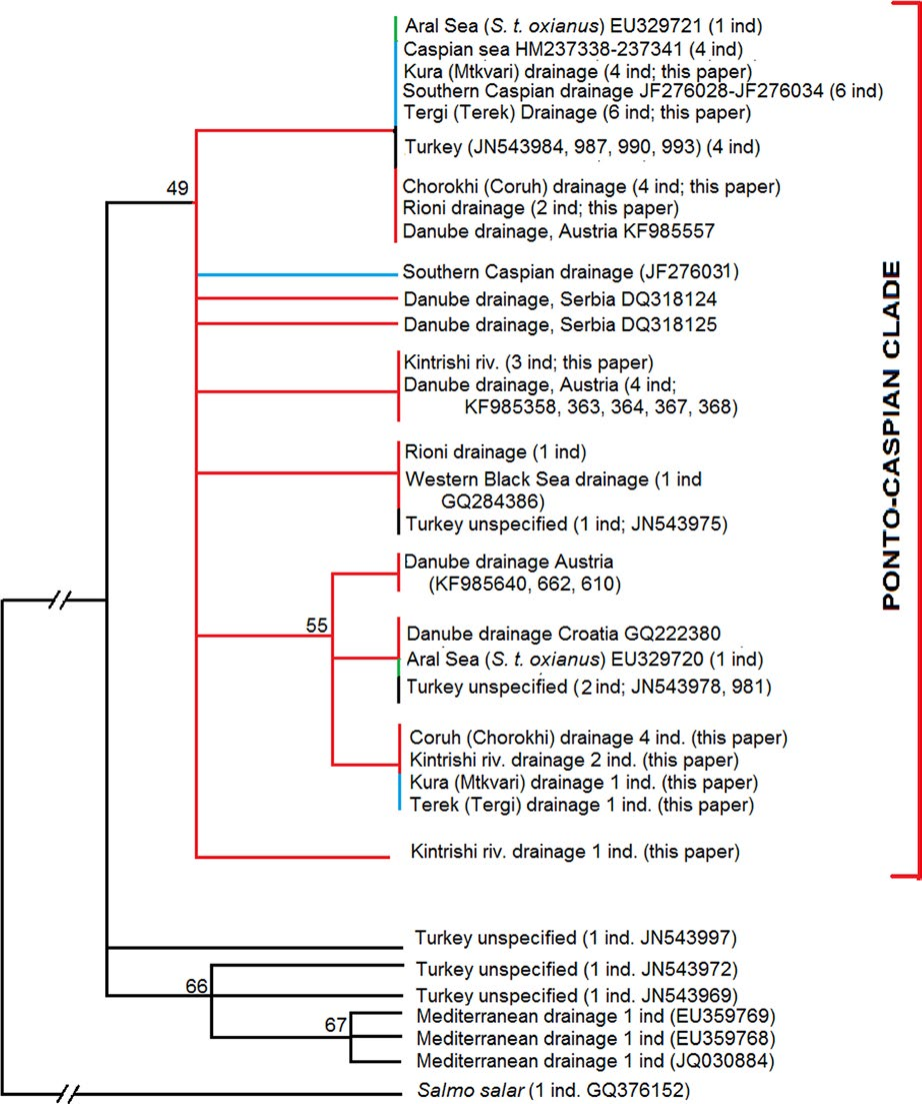
Maximum likelihood tree based on the control region sequences of *Salmo*. Bootstrap support shown above the nodes. Identical haplotypes shown separately if found at more than one location. Clades with bootstrap support below 49 shown as polytomies. Red branches—samples from the drainage of the Black Sea; blue branches—samples from the drainage of the Caspian Sea; green branches—samples from the drainage of the Aral Sea

The optimal substitution model for the ingroups included in the analysis was *T92* (Kumar et al., 2016). Both novel and downloaded sequences showed little variation within the studied fragment. However, all sequences from the drainages of Black and Caspian Seas were clustered in a monophyletic clade, albeit with modest bootstrap support, and related to trout from the Mediterranean (it was not possible to exactly identify locations for the sequences from Turkey). Remarkably, two sequences of *S. trutta oxianus* from the Aral Sea watershed belonged to the same clade. However, the sequences could not resolve phylogenetic relations of fish from the Ponto‐Caspian basin, and failed to separate Black Sea, Caspian, and Aral Sea lineages (Figure 6).

### 3.5 Morphometry

Principal component analysis based on the standardized residuals of 27 size‐removed body measurements extracted seven principal components with eigenvalues exceeding one. Cumulative weight of the components 1–4 exceeded 68% (Table 2a). Measurements showing relative height of fish bodies had the highest loadings on the first PC. Measurements of the head (relative length of snout) had the highest loading on the second PC. The distances in the hind part of the body had the highest loading on the third PC. Relative distances between the dorsal and adipose fins had the highest loadings on the fourth PC (Table 2b).

**Table 2:** The outcome of principal component analysis based on 28 size‐removed measurements of fish body (only trout from Georgia included). (a) Eigenvalues and % of variance for the PC exceeding one; (b) loadings of individual distances (see Figure 2 for details) on the first seven PC axes

(a)			
Component	Initial Eigenvalues		
	Total	% of Variance	Cumulative %
1	9.652	35.750	35.750
2	4.379	16.219	51.969
3	2.471	9.152	61.122
4	1.941	7.187	68.309
5	1.845	6.833	75.142
6	1.417	5.247	80.389
7	1.190	4.406	84.795

(b)							
Distances	Component						
	1	2	3	4	5	6	7
1–2	−0.109	0.893	−0.076	−0.050	0.143	0.186	0.027
1–3	0.150	0.746	0.227	−0.033	−0.204	−0.043	−0.254
3–4	0.511	−0.614	−0.098	0.197	0.464	0.023	−0.104
4–5	0.159	0.427	0.156	−0.086	−0.452	−0.577	0.004
5–6	−0.142	−0.006	0.392	−0.793	0.251	0.019	0.185
6–7	0.606	−0.312	−0.368	−0.021	−0.475	0.175	−0.139
7–8	−0.711	0.190	0.100	0.450	0.178	−0.055	0.242
8–9	−0.602	0.294	0.243	0.267	0.127	−0.063	0.293
9–10	0.280	0.272	−0.586	−0.554	−0.143	0.201	0.205
10–11	0.418	−0.067	0.481	0.294	−0.420	−0.259	0.088
11–12	−0.025	−0.320	0.390	0.259	−0.316	0.611	−0.023
12–13	0.626	−0.069	−0.496	0.086	0.290	−0.386	0.067
13–1	0.075	0.691	0.149	−0.139	0.202	−0.036	−0.547
3–13	0.741	0.442	0.138	0.170	−0.009	−0.036	−0.123
1–8	0.759	−0.234	−0.202	−0.028	0.428	−0.275	−0.026
3–12	0.835	−0.060	−0.075	0.361	0.155	0.021	0.018
4–13	0.923	0.150	0.158	0.079	0.077	−0.070	0.077
4–12	0.669	−0.125	0.506	−0.250	−0.052	−0.178	0.032
4–11	0.894	0.120	0.115	0.238	−0.040	0.108	0.058
5–12	0.764	−0.263	0.380	−0.165	0.217	0.120	0.087
5–11	0.523	−0.445	0.534	−0.292	0.064	0.124	0.150
5–10	0.712	0.500	0.134	0.122	0.046	0.024	0.287
6–11	0.775	0.345	−0.090	0.206	0.096	0.221	0.191
6–10	0.770	0.000	−0.296	−0.004	−0.247	0.231	−0.235
6–9	0.493	0.174	−0.340	−0.132	−0.381	−0.047	0.523
7–10	0.835	0.218	0.095	−0.162	0.080	0.080	−0.184
7–9	−0.071	0.751	0.002	0.040	0.337	0.333	0.154

**Figure 7:**
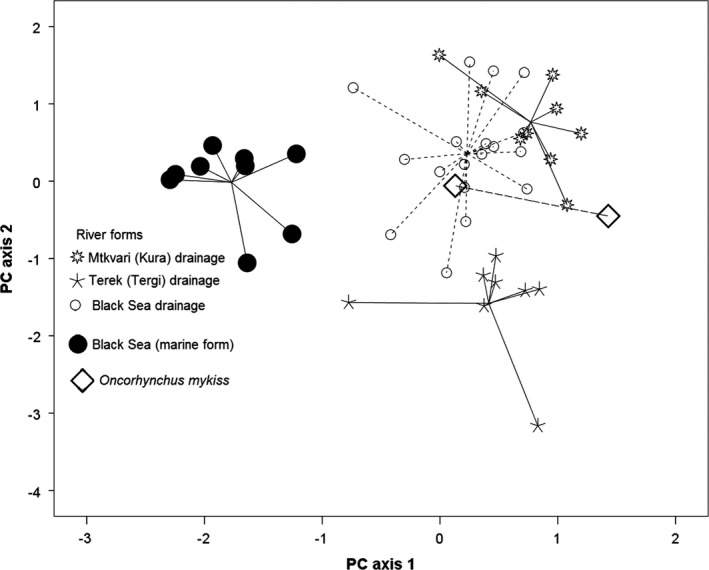
Individual scores of brown trout (river and marine forms) and naturalized rainbow trout from Georgian rivers and costal area of the Black Sea on the first and the second principal component axes based on 27 size‐removed body measurements (see Figure 2 for details)

The first PCA axis separated river trout (including rainbow trout) from the trout caught in the coastal area of the Black Sea (“Black Sea Salmon”). The second axis separated the fish from the basin of Terek (Tergi; nominal *S. ciscaucasicus*) from the rest of the samples (Figure 7). The third axis did not add any valuable information to the analysis, and the fourth axis separated rainbow trout (*Onychorhynchus mykiss*) from *Salmo* spp (result not shown). Simultaneously, the first and the second axes partly separated river trout from the Black and the Caspian (excluding Terek) watercourses (Figure 7).

The number of gill rakers at the first gill arch varied between 14 and 22, and did not show stable differences between the individuals from different populations, or between *Salmo* and *Oncorynchus* individuals. The two smallest individuals (body length 10 and 12 cm) had 14 rakers each, and the largest individual (28 cm) had 22 rakers; 30 individuals with intermediate body length (13–25 cm) had 16–18 rakers, the number coinciding with those reported by Turan et al. (2009) for *S. coruhensis*. Correlation between body length and the number of rakers was significant (*r* ^2^ = .81, *n* = 32, *p* < .01).

Of the 32 studied individuals, the majority had 10 rays in dorsal fins, and three individuals, two from the Caspian, and one from the Black Sea catchment, had nine rays. Finally, the number of rays in pectoral fin varied between 12 (12 individuals) and 13 (20 individuals), without respect to body size, genetics, or river catchment.

**Figure 8:**
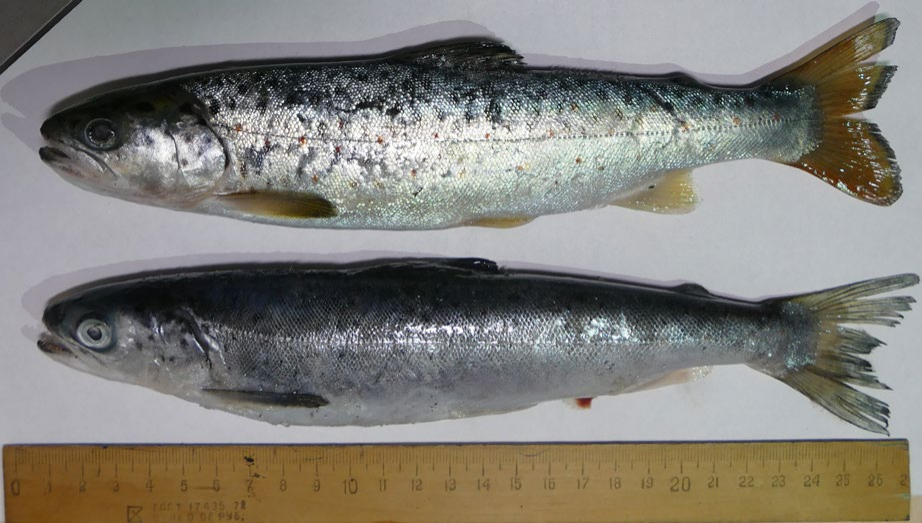
River form (upper image; Kintrishi catchment) and marine form (lower image; Black Sea coastal area near mouth of Kintrishi) of *Salmo labrax* from Black Sea drainage in Georgia; both individuals have identical mitochondrial haplotype

There are more or less stable differences in color pattern between the marine and freshwater forms of trout (Figure 8). The background was silver in marine form but yellowish in the freshwater trout. The individuals from Terek (Tergi) River were gray rather than yellowish. Red spots on the lateral side are paler in marine forms.

In conclusion, the studied qualitative characters did not help much to distinguish between individuals of different genetic or geographic groups, in contrast with body shape dimensions.

## 4 DISCUSSION

Brown trout from the Black and the Caspian Sea drainages comprises a monophyletic evolutionary lineage (matrilineal clade) distinct from brown trout from other parts of West Eurasia, including those from the drainages of Atlantic, Mediterranean, and the Indian Ocean. This lineage separated from other clades native to the rivers of Anatolia. Most of the subclades within this clade produce both riverine and anadromous forms, or may spontaneously switch between these two life modes. There are some differences in body proportions between different lineages of brown trout, although morphology should be used very carefully while differentiating among the nominal species: Our analyses suggest that body proportions and number of gill rakers, commonly used in taxonomy, may be associated with highly variable body size of the fish.

### 4.1 Diversification of brown trout and geological past

Basal diversification within *Salmo*, separating Atlantic salmon (*S. salar*) from the brown trout lineage occurred, according to different authors, from 9.6 to 15.4 mya (Alexandrou et al., 2013; Campbell, López, Sado, & Miya, 2013; Crête‐Lafrenière et al., 2012; Ma et al., 2015; Shedko, Miroshnichenko, & Nemkova, 2012). The split between the widely distributed European *S. trutta* and recently described species *S. ohridanus* and *S. obtusirostris* occurred 3 to 9 Mya, in the late Miocene or Pliocene (Crête‐Lafrenière et al., 2012; Macqueen & Johnston, 2014; see http://www.timetree.org/; Hedges et al., 2015 for review). If these dates are accepted, the Ponto‐Caspian lineage has been separated from the populations of the Anatolian rivers draining into the Persian Gulf and Mediterranean Sea ca. 2.4 mya, shortly after early Pleistocene glaciations began (Subcommission on Quaternary Stratigraphy, 2017). Consequently, all trout populations from the Black, Caspian, and Aral Sea drainages, from Austria to Iran and Kazakhstan, descend from this lineage dispersed during the Pleistocene, probably in Pleistocene interglacials, from the rivers of the northeastern Anatolia.

Schenekar et al. (2014) suggested that the divergence time between brown trout from the Atlantic and Black Sea basins in Austria occurred 320,000–745,000 years ago, based on the 1%–2% per MY divergence rate for *Thymalus* spp. (Koskinen, Haugen, Primmer, Schlotterer, & Weiss, 2002; Weiss, Persat, Eppe, Schlötterer, & Ublein, 2002). If the scale used by these authors is applied, the separation of the Ponto‐Caspian lineage should have happened only ca. 200 kya, hence in late Pleistocene. However, this dating would suggest the revision of the inferred divergence time for the entire genus *Salmo*, and we suggest there is not yet enough evidence for such revision.

It is likely that the divergence of Ponto‐Caspian trout was associated with glacial cycles. The landscape model of the last glacial maximum (Gavashelishvili & Tarkhnishvili, 2016) suggests that the river courses throughout the most of the Caucasus and Asia Minor were shorter than in present time, which means that the upper currents of these rivers would likely not be suitable for trout reproduction. A large continuous refugium was located at the southeastern coast of the Black Sea (Gavashelishvili & Tarkhnishvili, 2016; Van Andel & Tzedakis, 1996) and, hence, the brown trout populations could have survived there during glacial periods. The area at the southeastern coast of the Black Sea had climate and forest landscapes most similar to the interglacial period; trout habitats of that area (mostly in the basin of Coruh [Chorokhi] and other rivers draining into the Black Sea along its southeastern coast) were least affected by climatic fluctuations. This is the area of the highest genetic diversity of the Ponto‐Caspian trout, home also to the basal lineages of this clade. The split between this lineage and the Euphrates/Persian Gulf lineage happened in early Pleistocene. It is possible that some parts of the upper reaches of the Euphrates and Coruh (Chorokhi) changed their courses, causing isolation of some mountain populations, hence reinforcing the divergence of fish from the drainages of the Black Sea and the drainage of the Euphrates; a similar process explains the presence of two distinct lineages of trout in upper reaches of the Danube (Schenekar et al., 2014). Indeed, only the largest rivers of the northern Eurasia retained their courses through the entire Pleistocene (Shahgedanova, 2002).

The populations from the Black Sea were captured during the consequential glacial cycles in small isolated streams flowing into the southeastern part of the sea, and in interglacial periods, probably used the sea as a transit area, dispersing through different rivers of the same drainage. One of these lineages might gain some morphological peculiarities and lose ability to switch to anadromy, the form recently described as *S. rizeensis* (Turan et al., 2009). Other four major lineages have apparently maintained this ability. One of those dispersed into the Caspian drainage (and probably into the Aral Sea drainage), and three others remained in the Black Sea drainage. The most recent conjunction between the Black and Caspian Seas, through the lowland drainages north of the Caucasus Mountains, could have happened as late as 160 kya (Gelembiuk, May, & Lee, 2006) and even 9 kya (Reid & Orlova, 2002). However, over million years of isolation between the Black Sea lineages and the Caspian lineage of brown trout (according to a more likely estimate) suggest that the conjunction periods have been strongly reduced since earlier time. Although there are differences between average body proportions of trout from the Black and Caspian catchment (Figure 7), the morphological overlap is high. Morphological differences between the fish from the basin of Terek and Mtkvari (Kura) are stronger and fixed.

The split between the four Black Sea clades, including the Danube clade, occurred shortly after the initial split and could be associated with increasing time of glacial cycles and decreasing temperature during the glacial maxima in mid‐Pleistocene (Imbrie et al., 1993). In conclusion, Pleistocene glacial cycles and the latest transgressions between the Black and Caspian Seas are the main reasons of separation and genetic diversification of Ponto‐Caspian lineages of brown trout.

### 4.2 Riverine and lacustrine versus anadromous mode of life—obligatory or facultative?

It has never been established to what extent anadromy is an inheritable feature of individual evolutionary lineages of brown trout. Even in better studied American rainbow trout, there are multiple gaps of knowledge concerning this question (Kendall et al., 2015). In the earliest treatises (e.g., Sabaneev, 1875) Atlantic salmon and Black Sea salmon were clumped together under the name *Salmo salar*, although the author mentioned that the salmon from the Caspian Sea drainage might belong to a different species, Caspian salmon (*S. caspius*). Conversely, for brown trout from the rivers and streams of Europe and the Caucasus, including drainages of the Black and Caspian Seas, Sabaneev (1875) used the name *Salmo fario*, considering it a purely freshwater form.

Field studies conducted in 1920s revealed that the transition between the riverine and anadromous life mode occurs easily. Berg (1959) suggested that an individual trout may occasionally change the riverine life mode to an anadromous one during its life cycle. However, the question remains—to what extent is anadromy a genetic feature typical for only some lineages of brown trout? It has been shown for other salmonids, for example, *Oncorhynchus mykiss* (Kendall et al., 2015; Nichols, Edo, Wheeler, & Thorgaard, 2008) and *S. salar* (Perrier, Bourret, Kent, & Bernatchez, 2013) that change between the life modes is associated with a complex genetic mechanism, and these are heritable. Turan et al. (2009) showed that within the basin of Coruh (Chorokhi), the two forms coexist, forms which he named *S. rizeensis* and *S. coruhensis*, the former purely riverine and the latter anadromous. This study suggested that, indeed, the mitochondrial haplogroup of *S. rizeensis* was never found in fish caught in the Black Sea. Simultaneously, the individuals from the Black Sea (as well as a single Caspian salmon included in this analysis), although they differ from the freshwater fish morphologically (Figures 7 and 8), belong to various branches within a monophyletic Ponto‐Caspian clade, which has *S. rizeensis* as the sister clade. Anadromous life mode presents in brown trout from the catchment of Atlantic Ocean (*S. trutta* s. str.). It is unknown for *S. marmoratus* and Anatolian *S. platycephalus* (most closely related to the Ponto‐Caspian forms)*,* Balkan *S. ohridanus,* or *S. obtusirostris*. The absence of anadromy of these forms may be related to a high salinity of the seas connecting to the rivers populated by these species (Thunell & Williams, 1989). We hypothesize that the reappearance of anadromy in brown trot from the Black Sea and Caspian catchments is related with low salinity of these seas.

### 4.3 Taxonomic inference

The trout from the Caspian Sea basin (Caspian trout) is a monophyletic lineage. Nominal *S. caspius* from the drainage of Mtkvari (Kura) and southern Caspian, on one hand, and nominal *S. ciscaucasicus* from the Terek (Tergi) River are monophyletic. Moreover, the river forms of this fish are morphologically distinct from each other: Nominal *S. ciscaucasicus* has shorter snout, and this feature is nearly diagnostic. Black Sea trout have on average shorter trunk than nominal *S. caspius*, and narrower body, although these differences cannot be used for diagnosis between the two lineages.

We suggest using the valid names *S. caspius* for brown trout from the rivers flowing into the Caspian Sea from the south and southwest, and *S. ciscaucasicus* for that found in the Terek River and other rivers flowing into the Caspian from the north and northwest. Meanwhile, the taxonomic position of lacustrine *S. ischchan* and *S. ezenami* needs further analysis.

The anadromous individuals from the Black Sea catchment, described as *S. coruhensis* by Turan et al. (2009), were shown not to represent a monophyletic lineage with respect to both the riverine and anadromous fish from Georgia, as well as to the fish from the Danube drainage. Turan et al. (2009) showed some morphological differences between the anadromous fish from Coruh catchment and *S. labrax* from the northern part of the Black Sea drainage. However, the latter were not investigated genetically, and their characteristic features (lower number of gill rakers and different body measurements) may be associated with body size of fish, smaller than that of other nominal species. The number of rakers may increase with body size of fish (Ross, Martínez‐Palacios, Aguilar Valdez, Beveridge, & Chavez Sanchez, 2006), and moreover, running PCA without excluding size would obviously harvest the first component dependent largely on body size (Thorpe & Leamy, 1983), an extremely variable characteristic in fish. Hence, there is not sufficient evidence of morphological separation between *S. labrax* and *S. coruhensis*. Consequently, we suggest a conservative approach, and all individuals from the watercourses of Coruh (Chorokhi), Kintrishi, Rioni, Enguri, Danube, and those caught in the Black Sea (except *S. rizeensis*, a narrow‐ranged purely riverine form) we consider conspecific at this stage, and use the priority name *S. labrax* (Black Sea salmon).

We should emphasize the findings reported here are based on sequences from a mitochondrial cytochrome *b* fragment, and might be refined if nuclear sequence data were added to the analysis. However, based on a limited number of existing studies that combine nuclear and mitochondrial sequence data from salmonids, mitochondrial and nuclear sequences converge on the same clades in genus *Salmo* (Crête‐Lafrenière et al., 2012). Simultaneously, one can expect patterns of incomplete lineage sorting and gene flow between closely related lineages, similar to that revealed in Central Europe as a result of microsatellite genotyping (Schenekar et al., 2014), and obviously further analysis including improved sampling and more DNA markers will substantially improve our knowledge of western Eurasian trout and salmon phylogeny and population structure.

## CONFLICT OF INTEREST

None declared.

## AUTHOR CONTRIBUTIONS

LN conducted the field work, processed the samples for molecular genetic analysis, and together with DT analyzed phylogenies. DT and LN designed the study and prepared the manuscript. EG conducted morphometric analysis and assisted in DNA processing.
